# Japanese giant benign prostatic hyperplasia: Sibling cases

**DOI:** 10.1002/iju5.12467

**Published:** 2022-05-09

**Authors:** Takehiro Ohyama, Fumiyasu Endo, Masaki Shimbo, Kazunori Hattori

**Affiliations:** ^1^ Department of Urology St. Luke's International Hospital Tokyo Japan

**Keywords:** family history, giant benign prostatic hyperplasia, holmium laser enucleation of the prostate

## Abstract

**Introduction:**

The pathophysiology of benign prostatic hyperplasia (BPH) remains incompletely understood but is likely multifactorial. Inflammation and metabolic factors may increase the risk of BPH. Several studies have evaluated the possible roles played by genetic factors. Here, we describe two cases of suspected familial BPH.

**Case presentations:**

We report the cases of two brothers, aged 77 and 69 years, with giant BPH. As both exhibited urinary retention, we performed Holmium Laser Enucleation of the Prostate (HoLEP) and obtained tissue samples weighing 276 g and 153 g, respectively. The postoperative courses were good.

**Conclusion:**

We experienced two cases of sibling BPH with volumes exceeding 200 mL and successfully treated them with HoLEP.

**Key message**

We report the cases of two brothers with giant BPH. We used HoLEP to treat both patients.

## Introduction

Medical records indicate that nearly 70% of males in the United States aged 60 to 69 years, and almost 80% of those aged ≥70 years, have some degree of benign prostatic hyperplasia (BPH),^1^ often associated with chronic and progressive lower urinary tract symptoms or complications; many men seek surgical treatment. Prostatic enlargement caused by BPH rarely exceeds 100 g; more severe BPH occurs in only 4% of men older than 70 years.^2^ Giant BPH is defined as a prostate weight >200 g or >500 g; the lower threshold was suggested by Japanese authors,^3^ probably because giant BPH is rare in Asian countries compared to other countries.^4^ The pathophysiology of BPH remains incompletely understood but is likely multifactorial; inflammation and/or metabolic factors may play roles. Although studies of familial trends in BPH and genetic works^5^, ^6^ have indicated that genetic factors may indeed be in play, their nature, particularly in patients with giant BPH, remains unclear. Here, we report the cases of two brothers with giant BPH.

## Case presentations

**Fig. 1:**
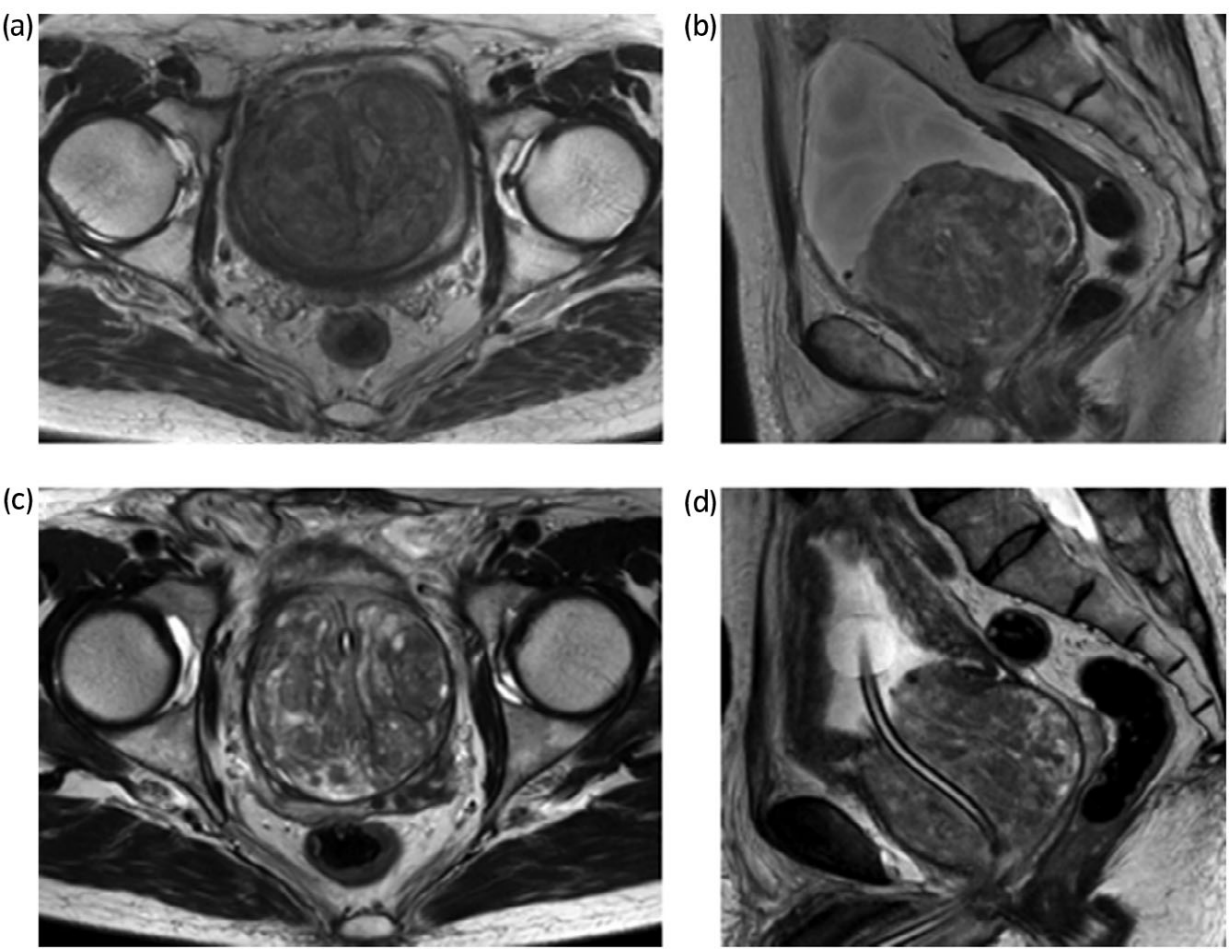
(a) An axial section of an MRI T2‐weighted image. (This is a 77‐year‐old case.) (b) A sagittal section of an MRI T2‐weighted image. (This is a 77‐year‐old case.) (c) An axial section of an MRI T2‐weighted image. (This is a 69‐year‐old case.) (d) A sagittal section of an MRI T2‐weighted image. (This is a 69‐year‐old case).

We describe the cases of two brothers, aged 77 and 69 years, with giant BPH. One 77‐year‐old patient had engaged in intermittent urethral self‐catheterization, but repeated urinary tract infections encouraged him to request surgery. The 69‐year‐old patient had exhibited high PSA levels long term, and requested surgery because of urinary retention and a need for urethral catheterization. Their prostate‐specific antigen (PSA) levels were high (23.38 and 30.89 ng/mL, respectively), but magnetic resonance imaging (MRI) revealed only enlarged prostates with no suspicion of cancer (9.8 × 8.2 × 9.0 cm, estimated volume 376 mL [Fig. 1a,b] and 7.6 × 7.6 × 7.8 cm, estimated volume 234 mL [Fig. 1c,d], respectively). The PSA densities were 0.06 and 0.13 respectively. The patients' father lacked any history of BPH, but the paternal grandfather had complained of dysuria, which might have indicated BPH. We performed holmium laser enucleation of the prostate (HoLEP). Totals of 276 g and 153 g of tissue were removed during single‐step surgeries. The enucleation times were 41 and 69 min. The morcellation times were 140 and 31 min, with morcellation efficacies of 1.97 and 4.94 g/min, respectively. The postoperative hemoglobin (Hb) decreases were 1.4 and 0.2 g/dL, respectively.

The postoperative courses were good, and the catheters were removed on the second and third days, respectively. Although slight urinary incontinence was evident, the patients were discharged with good urinary status; the urine volume was about 200 mL per urination. Pathological examination revealed hyperplasia. At 6 months after surgery, their urinary status remained good, and their PSA levels were 0.17 and 2.35 ng/mL, respectively.

## Discussion

We report the cases of two brothers aged 77 and 69 years diagnosed with giant BPH.

The cause of giant BPH remains uncertain, but familial BPH tends to be larger than sporadic BPH.^5^ Giant BPH is quite rare; if a sibling also evidences giant BPH, a genetic relationship is strongly inferred. Although our cases might have revealed genetic associations, we did not perform genetic testing. No dedicated, commercial genetic test for familial BPH is yet available, but some reports have indicated that genetic variants of *GATA3* may increase the susceptibility to, and etiology of, inheritable BPH.^6^ In the future, when encountering a patient with giant BPH, it will be important to interview the family and perform genetic analysis. Such work will shed light on the pathogenesis of giant prostate enlargement and encourage the development of novel therapies. It has been reported that familial BPH patients often develop BPH at a young age^5^; it would be appropriate, therefore, to examine such young people medically.

**Fig. 2:**
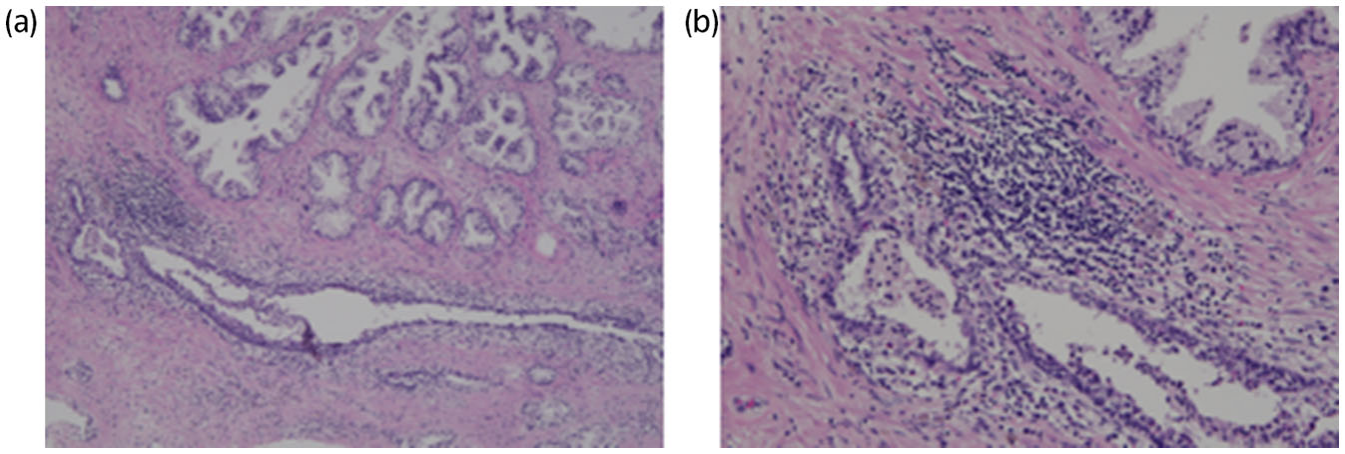
This is a 77‐year‐old case's findings. (a) This image reveals glandular enlargement of the prostate (hematoxylin and eosin staining [H&E] × 4). (b) This image reveals lymphocytic infiltration, reflecting inflammation (H&E × 20). [Colour figure can be viewed at wileyonlinelibrary.com]

The pathogenesis of BPH remains unclear, but it has been speculated that BPH develops when the balance between cell proliferation and death is disrupted and proliferation exceeds death.^7^ Androgens, estrogens, epithelial–stromal interactions, and growth factors are involved in the pathogenesis of BPH,^8^, ^9^ as are other factors such as inflammatory cells.^10^ The combined effects of these factors seem to cause significant prostate enlargement. In our cases, pathological examination revealed both inflammatory cell infiltration and interstitial hyperplasia (Fig. 2a,b. The pathological profiles were similar; we thus present the details of only the 77‐year‐old case.)

Although an open procedure is an option for giant BPH according to the guidelines,^11^ HoLEP is usually performed in our hospital because open prostatectomy is invasive and causes a great deal of bleeding. Based on the favorable results achieved in our hospital, HoLEP was the treatment selected for these two cases.

When performing HoLEP for large BPH, the most difficult procedure is morcellation. The efficiency thereof is low, especially for large prostates (>200 g)^12^; second‐stage surgery is often required to avoid complications such as hypothermia, which can occur because the operative time is long. One review found that the efficiency of morcellation was 3.95 g/min^13^, but in our one case, the efficiency was significantly lower at 1.97 g/min. However, this was countered by the fact that enucleation was rapid because the surgeon was very experienced in HoLEP. The enucleation efficacies were 6.73 and 2.22 g/min, well above the 2 g/min reported in the past.^12^ The postoperative decrease in Hb levels was tolerable, indicating once again that HoLEP usefully treats giant prostates.

## Conclusion

We successfully performed one‐step HoLEP and removed more than 200 g of BPH from each of the two patients. As familial cases of BPH are few in number, it is important to explore any family history of BPH.

## Author contributions

Takehiro Ohyama: Data curation; project administration; writing – original draft; writing – review and editing. Fumiyasu Endo: Conceptualization; supervision; writing – review and editing. Masaki Shimbo: Supervision; writing – review and editing. Kazunori Hattori: Supervision.

## Conflict of interest

None declared.

## Approval of the research protocol by an institutional reviewer board

No.

## Informed consent

No.

## Registry and the registration no. of the study/trial

No.
